# Frailty inclusive care in acute and community-based settings: a systematic review protocol

**DOI:** 10.1186/s13643-021-01638-0

**Published:** 2021-03-26

**Authors:** Carmel L. Montgomery, Gareth Hopkin, Sean M. Bagshaw, Erin Hessey, Darryl B. Rolfson

**Affiliations:** 1 Institute of Health Economics, 1200 10405 Jasper Avenue, Edmonton, Alberta T5J 3N4 Canada; 2 Department of Critical Care Medicine, Faculty of Medicine and Dentistry, University of Alberta, 2-124 Clinical Sciences Building, 11350 83 Ave, Edmonton, Alberta T6G 2G3 Canada; 3 Critical Care Strategic Clinical Network, Alberta Health Services, Edmonton, Alberta Canada; 4 Division of Geriatric Medicine, Department of Medicine, Faculty of Medicine and Dentistry, University of Alberta, 1-198 Clinical Sciences Building, 11350 83 Ave, Edmonton, Alberta T6G 2G3 Canada

**Keywords:** Frailty, Multi-component interventions, Cost effectiveness

## Abstract

**Background:**

Frailty is a known risk factor for an array of adverse outcomes including more frequent and prolonged health services use and high health care costs. Aging of the population has implications for care provision across the care continuum, particularly for people living with frailty. Despite known risks associated with frailty, there has been limited research on care pathways that address the needs of persons living with frailty. Our study aims to review and examine, in a rigorous way, the quality of evidence for multi-component interventions and care pathways focused on frailty.

**Methods:**

A comprehensive electronic search strategy will be used to identify studies that evaluate multi-component interventions or care pathways for persons living with frailty. The search strategy will include terms for frailty, multi-component interventions, effectiveness, and cost effectiveness applied to the following databases: MEDLINE (OVID), EMBASE (OVID), CINAHL (EBSCO), Cochrane Central Register of Controlled Trials (CENTRAL), and Cochrane Database of Systematic Reviews. An adapted search for Google Scholar and gray literature databases will also be used. References of included studies will be hand-searched for additional citations of frailty-inclusive care. Known experts and corresponding authors of identified articles will be contacted by email to identify further eligible studies. Risk of bias will be assessed using the Effective Public Health Practice Project Quality Assessment tool. Data will be extracted from eligible studies and it is anticipated that narrative analysis will be used. If studies with sufficient homogeneity are found, then pooled effects will be reported using meta-analysis.

**Discussion:**

This review will appraise the evidence currently available on multi-component frailty interventions. Results will inform on clinical pathway development for people living with frailty across the care continuum and will guide future research to address gaps in the literature and areas in need of further development.

**Systematic review registration:**

PROSPERO CRD42020166733

**Supplementary Information:**

The online version contains supplementary material available at 10.1186/s13643-021-01638-0.

## Background

The general health and functional status of patients prior to acute illness and other stressors are accepted as important determinants of hospital outcomes [1, 2]. This status can be captured by measuring frailty, an age-related state of increased vulnerability with disproportionate changes in health status in response to stressors [3]. This creates the opportunity to develop and implement care protocols that are tailored to the health needs of persons who live with frailty.

As the worldwide population ages, the associated increase in prevalence of frailty across the care continuum presents urgent challenges to providing consistently appropriate care. Recent findings from cohort studies have shown prevalence of frailty in the Canadian adult community population is 8-24%, increasing as age advances from 2% (18-34 years) to 20% (≥65 years) [4, 5]. Frailty is common in assisted living environments (29%), and among adults admitted to ICU (28-32%) [6–9]. Frailty in hospitalized patients has been associated with gradient increases in hospital mortality, intensity of organ support, frequency and duration of health services use, and cost, when compared to non-frail patients [8–10].

Despite knowledge that frailty presents high risk for suboptimal outcomes, development and evaluation of comprehensive frailty interventions appears limited to specific circumstances such as improving patient flow after surgery (enhanced recovery after surgery [ERAS]), addressing frailty-related diseases or disability (e.g., delirium, dementia, depression), and providing focused interventions (e.g., nutrition, exercise) in acute and community-based care settings. The opportunity to view frailty as a multi-system syndrome is often overlooked by generic pathways where aspects of frailty are entirely unnoticed or assumed to be addressed by broad criteria such as chronological age. Frailty inclusive care, as defined herein, is an approach to care in any setting that starts with authentic frailty case-finding, followed by further assessment of the underlying vulnerabilities (components), and then leading to advance care planning, general measures to prevent or slow progression, and specific steps to address frailty components. Frailty inclusive care interventions may be generic, or may be specific to medical condition or care setting.

The primary aim of this project is to rigorously examine and document the quality of evidence for multi-component interventions (e.g., frailty-inclusive care pathways) encompassing the broader patient journey through both community and hospital care. In so doing, we will be mindful of a variety of frailty constructs and measures, and the ways that frailty inclusive care has been operationalized using different frailty assessment measures, evaluated based on any comparators and outcomes across patient populations. This will directly inform further development and implementation of frailty-specific recommendations as part of a larger program of work in this area.

## Methods/design

The full systematic review protocol is registered with the International Prospective Register of Systematic Reviews (PROSPERO) (ID CRD42020166733, April 28, 2020). A recent search of PROSPERO and the Cochrane Library indicated there were no registered reviews focused on the proposed topic. A Preferred Reporting Items for Systematic Reviews and Meta-Analyses Protocols (PRISMA-P) checklist is included as an additional file [11] (Additional file 1).

### Search methodology and screening

The search strategy will be developed by an information specialist, in partnership with the broader review team and peer-reviewed by a second information specialist. The initial search strategy will be translated for use across bibliographic databases and web search engines. We will identify potentially relevant studies by searching the following bibliographic databases from 2000 onwards (i.e., when frailty became a term used in research) in all languages, using a combination of keywords (i.e., free text) and MeSH terms: MEDLINE (OVID), EMBASE (OVID), CINAHL (EBSCO), Cochrane Central Register of Controlled Trials (CENTRAL), and Cochrane Database of Systematic Reviews (CDSR) (Table 1).

**Table 1:** Search strategy—Medline

#	Searches
1	Frail Elderly/
2	Frailty/
3	Geriatric Assessment/
4	Sarcopenia/
5	(frail* or nonfrail*).tw,kf.
6	(Fried* adj2 (definition or index or phenotyp* or scor*)).tw,kf.
7	(functional-status and (low or poor*)).tw,kf.
8	(geriatric adj2 (assess* or evaluat* or screen*)).tw,kf.
9	sarcop?eni*.tw,kf.
10	or/1-9 [MeSH & Keywords for Frailty]
11	Clinical Protocols/
12	Critical Pathways/
13	Enhanced Recovery After Surgery/
14	Patient Care Planning/
15	((care or clinical or critical) adj protocol*).tw,kf.
16	(enhanced-recovery adj2 (path* or program* or protocol* or surg*)).tw,kf.
17	eras.tw,kf.
18	erp*.tw,kf.
19	(fasttrack* or fast-track*).tw,kf.
20	((multicomponent or multi*-component* or multidimens* or multi-dimens* or multimodal or multi-modal) adj2 (intervention* or program*)).tw,kf.
21	pathway*.tw,kf.
22	(rapid* adj2 recover*).tw,kf.
23	or/11-22 [MeSH & Keywords for Pathways]
24	10 and 23
25	*economics/
26	exp *"Costs and Cost Analysis"/
27	(economic adj2 model*).mp.
28	(cost minimi* or cost-utilit* or health utilit* or economic evaluation* or economic review* or cost outcome or cost analys?s or economic analys?s or budget* impact analys?s).ti,ab,kf,kw.
29	(cost-effective* or cost-benefit or costs).ti,kf,kw.
30	(life year or life years or qaly* or cost-benefit analys?s or cost-effectiveness analys?s).ab,kf,kw.
31	(cost or economic*).ti,kf,kw. and (costs or cost-effectiveness or markov).ab.
32	or/25-31 [Adapted from the CADTH Narrow Economic Filter]
33	randomized controlled trial.pt.
34	clinical trial.pt.
35	randomi?ed.tw.
36	randomly.tw.
37	trial.tw.
38	groups.tw.
39	or/33-38 [Adapted from the Cochrane Filter for RCTs]
40	(pretest* or pre-test*).tw,kf.
41	pre-post-test*.tw,kf.
42	(quasiexperimental or quasi-experimental).tw,kf.
43	repeated-measur*.tw,kf.
44	time-series.tw,kf.
45	(patient? or hospital$).hw. and (study or studies).ti,hw.
46	(controlled adj3 before adj3 after).tw.
47	((before adj10 (after or during)) and control).tw.
48	or/40-47 [MeSH & keywords for quasiexperimental design]
49	24 and (32 or 39 or 48)
50	limit 49 to yr="2000 -Current"
51	remove duplicates from 50

Other searches and strategies will include Google Scholar; ProQuest Dissertation and Theses Global; World Health Organization (WHO) International Clinical Trials Registry Platform (ICTRP) and clinicaltrials.gov for ongoing trials; published abstracts; and conference proceedings for 2 years prior to search date. An a priori list of websites which may contain eligible studies that have not been indexed will also be reviewed (Table 2).

**Table 2:** Web sites for non-indexed study publications

https://www.cfn-nce.ca/	
https://frailty.net/	
https://frailty-sarcopenia.com/	
https://anzssfr.org/	
https://www.bgs.org.uk/	
https://www.americangeriatrics.org/	
https://thecanadiangeriatricssociety.wildapricot.org/	
https://www.eugms.org/home.html	

Citations identified by the searches will be de-duplicated in EndNote (Clarivate Analytics, v.9.), then uploaded to a Covidence (Veritas Health Innovation, Melbourne, Australia) library. Studies reported in languages other than English will be translated to English using Google Translate [12]. Two reviewers will independently screen the titles and abstracts of studies identified by the search (i.e., primary screening). Studies that meet the predetermined eligibility criteria from title and abstract review will undergo screening of full text (i.e., secondary screening) by two independent reviewers. Any disagreements will be resolved by consensus or the decision of a third party.

### Study selection

We will include studies in any clinical setting that use an established frailty measure for case-finding. To improve consistency, a table of 93 eligible frailty measures was compiled from 13 literature reviews over the past decade, and classified based on the model of frailty (i.e., physical frailty, cumulative deficit, multidimensional, geriatric syndromes) and the method of administration (i.e., clinician-report, clinician-judgment, self-report, performance-based, anthropometric, calculated) [13–25] (Table 3). To qualify as multi-component, more than one intervention must be present, targeting different components of frailty. The multi-component intervention may include advance care planning, general measures to prevent or slow progression (e.g., a combined exercise and nutritional program), and detailed steps to address frailty. Specific interventions responding to components of frailty identified in particular individuals may include problems with cognition, mood, balance and mobility, continence, medications, and social support.

**Table 3:** Valid frailty measures

Measure	Frailty model	Scoring method
11 item Frailty Index [26]	Multidimensional	Predefined fields
5 item Frailty Index (mFI) [27]	Multidimensional	Predefined fields
Balance and Muscle Strength [28]	Physical frailty	Functional performance and self-report
Beaver Dam Eye Study Measure [29]	Physical frailty	Performance
Brief Clinical instrument to Classify Frailty [30]	Multidimensional	Clinician report
Brief Frailty Index (bFI) [31]	Multidimensional	Self-report and performance
Brief Risk Identification of Geriatric Health Tool (BRIGHT) [32]	Multidimensional	Self-report
British Frailty Index [33]	Cumulative deficit	Predefined fields
Care Partner derived Frailty Index based on CGA (CP-FI-CGA) [34, 35]	Cumulative deficit	Care partners using predefined fields
Chair stands [36]	Physical frailty	Performance
Chinese Canadian Study of Health and Aging Clinical Frailty Scale Telephone Version [37]	Multidimensional	Judgment-based
Clinical Frailty Scale (CFS) [5]	Multidimensional	Judgment-based
Clinical global impression of change in physical frailty (CGIC-PF)	Cumulative deficit	Predefined fields
Comprehensive Assessment of frailty [38]	Physical frailty	Performance, self- and clinician report
Comprehensive frailty assessment instrument (CFAI) [39]	Multidimensional	Self-report
Continuous Composite Measure of Frailty [40]	Physical frailty	Performance, self-report
Continuous frailty scale [41]	Physical frailty	Self-report and Performance
EASYcare - short version [42]	Multidimensional	Clinician report
EASY-Care Two-step Older persons Screening—Easy-Care TOS [43]	Multidimensional, CGA	Clinician report
Edmonton Frail Scale (EFS) [44]	Multidimensional, CGA	Functional performance, self-report, and clinician report
Electronic Frailty Index (eFI) [45]	Cumulative deficit	Predefined fields
Emergency General Surgeries Frailty Index–EGS-FI [46]	Multidimensional	Clinician report
Evaluative Frailty Index for Physical Activity (EFIP) [47]	Cumulative deficit	Clinician report
Fatigue, Resistance, Ambulation, Illness, Loss of Weight (FRAIL) [48]	Physical frailty	Self-report
Forced Expiratory Volume (FEV1) [49]	Physical frailty	Performance
Frail Non Disabled (FIND) [50]	Physical frailty	Self-report
FRAIL–Frailty and Autonomy Scoring Instrument of Leuven [51]	Multidimensional	Self-report
Frailty Assessment for Care Planning Tool (FACT) [52–54]	Multidimensional, CGA	Clinician report
Frailty GIR Evaluation (FRAGIRE) [55]	Multidimensional	Clinician report
Frailty Index (FI) [56, 57]	Cumulative deficit	Predefined fields
Frailty index derived from comprehensive geriatric assessment (FI-CGA) [58]	Cumulative deficit, CGA	Trained specialists using predefined fields
Frailty Index for Elders (FIFE) [59]	Multidimensional	Clinician report
Frailty Phenotype, CHS index [60]	Physical frailty	Self-report and performance
Frailty predicts death One yeaR after Elective CArdiac Surgery Test (FORECAST) [61]	Physical frailty	Performance, self-, and clinician report
Frailty Screening Questionnaire (FSQ) [62]	Physical frailty	Self-report
Frailty Screening Tool [63]	Multidimensional, CGA	Self- and clinician report
Frailty Trait Scale (FTS) [64]	Physical frailty	Performance
Functional Ability Index (FA Index) in the LUCAS Cohort [65]	Physical frailty	Self-report
Functional assessment screening package [66]	Multidimensional	Predefined criteria
G-8 Geriatric Screening Tool [67]	Multidimensional	Self-report
G8 Questionnaire [68]	Multidimensional, CGA	Clinician report
Gait Speed Test [GST] [69]	Physical frailty	Performance
Geriatric Functional Evaluation (GFE) [70]	Multidimensional	Self-report
Gérontopôle Frailty Screening Tool (GFST) [71]	Physical frailty	Clinician report
Groningen Frailty Indicator (GFI) [72]	Multidimensional	Self-report
Guilley Frailty Instrument [73]	Multidimensional	Self-report
Hand grip strength [74]	Physical frailty	Performance
Health Status Form– HSF [75]	Multidimensional	Self-report
Identification of Seniors at Risk (ISAR) [76]	Multidimensional	Self-report
Inactivity and Weight Loss [77]	Physical frailty	Clinician report
Índice de Vulnerabilidade Clínico-Funcional IVCF-20 [78]	Multidimensional, CGA	Self-report
Instrumento Multidimensional de rastreio da Sindrome da Fragilidade (IMSIFI) [79]	Multidimensional, CGA	Clinician report
INTER-FRAIL Study Questionnaire [80]	Multildimensional, CGA	Self-report
Kaigo-Yobo CheckList [81]	Multidimensional, CGA	Self-report
Kihon Check-list (KCL) [82]	Multidimensional, CGA	Self-report
KLoSHA Frailty Index [83]	Multidimensional, CGA	Clinician report
Korean Frailty Index [84]	Multidimensional, CGA	Clinician report
Margliano-Cacciafesta polypathological scale (MCPS) [85]	Multidimensional	Clinician report
Modelo Fried adaptado [86]	Physical frailty	Self-report
Modified Frailty Score [87]	Multidimensional	Performance
Modified Physical Performance Test (mPPT) [88]	Physical frailty	Performance
Multidimensional Prognostic Index (MPI) [89]	Multidimensional, CGA	Clinician-report
Opasich Frailty Measure [90]	Physical frailty	Performance
Physical frailty score [91]	Physical frailty	Performance
Predictive Physical Frailty Score [92]	Physical frailty	Self- and clinician report, performance
PRISMA-7 [93]	Multidimensional	Self-report
Prognostic Frailty Risk Score [94]	Multidimensional, CGA	Clinician report
Prognostic Frailty Score [95]	Multidimensional, CGA	Self-report and performance
Puts Frailty Criteria [96]	Multidimensional	Self-report and performance
Rapid Geriatric Assessment (RGA) [97]	Physical frailty	Self-report
Reported Edmonton Frail Scale (REFS) [98]	Multidimensional, CGA	Clinician and self-report
Resident Assessment Instrument, Minimum Data Set (RAI-MDS) [99]	Multidimensional, CGA	Clinician report and performance
Rothman Frailty Criteria [100]	Multidimensional	Self-report and performance
Schoevaerdts Index [101]	Multidimensional, CGA	Clinician report
SEGAm–Modified Short Emergency Geriatric Assessment [102]	Multidimensional, CGA	Clinician report
Self-report Screening Tool for Frailty [103]	Physical frailty	Self-report
Self-rated Health Deficits Index (HDI) [104]	Multidimensional	Self-report
SHARE Frailty Instrument [105]	Physical frailty	Self-report, calculator
SHARE Frailty Instrument 75+ [106]	Physical frailty	Self-report, calculator
Sherbrooke Postal Questionnaire (SPQ) [107]	Multidimensional, CGA	Self-report
Short physical performance battery (SPPB) [108]	Physical frailty	Performance
Strawbridge questionnaire [109]	Multidimensional	Self-report
Study of Osteoporotic Fractures (SOF) Index [110]	Physical frailty	Self-report and performance
Subset of Functional Status Questionnaire [111]	Multidimensional	Self-report
Three-City Study Frailty Criteria [112]	Multidimensional	Performance
Tilburg Frailty Indicator (TFI) [113]	Multidimensional, CGA	Self-report and performance
Timed Up and Go (TUG) [114]	Physical frailty	Performance
Trauma-Specific Frailty Index (TSFI) [115]	Cumulative deficit	Predefined fields
Triage Risk Screening Tool [116]	Multidimensional, CGA	Clinician report
Upper Extremity Function (UEF) Frailty [117]	Physical frailty	Performance
Vulnerable Elders Survey (VES-13) [118]	Multidimensional	Clinician report
Winograd Screening Instrument [119]	Physical frailty	Clinician report
Women’s Health Initiative Observational Study (WHI-OS) Multicomponent Measure [120]	Physical frailty	Self-report and performance

### Eligibility criteria

#### Population

The population of interest is all adult patients (≥18 years) defined as living with frailty, assessed by a validated frailty measurement, in acute, intermediate, or community-based care settings.

#### Intervention

We will identify multi-component interventions that have been developed and evaluated for their impact on individuals living with frailty, and the health services that comprise their care. Any intervention that can be applied to the broader patient journey through both community and hospital care will be included. Examples of interventions of interest include enhanced recovery after surgery (ERAS), multi-component fast track surgery programs or “prehabilitation,” hospital to community transition processes, or care pathways that identify frailty and trigger comprehensive geriatric assessment (CGA) to address components of frailty. Interventions (e.g., pharmacological treatments, rehabilitative therapies, nutritional counseling) that are implemented independently or do not influence the overall care plan will be excluded, unless they are used in combination. Screening for frailty without intervention or CGA without the use of frailty measures to inform a personalized assessment or care plan will be excluded.

#### Comparisons

All comparisons will be included. We anticipate that in most cases usual care within hospital and/or the community will be compared to the intervention.

#### Outcomes

Outcomes include those associated with evaluation of multi-component frailty care implementation, including process measures (e.g., measures of fidelity; acceptability; feasibility) targeting any person living with frailty, health service utilization (i.e., readmission, contact with services), health outcomes (e.g., adverse events, morbidity, mortality, institutionalization), economic outcomes (e.g., cost, cost-effectiveness), and patient-oriented measures (e.g., quality of life, well-being, satisfaction with care, caregiver burden).

#### Study designs

We will consider all randomized trials (e.g., patient level or cluster), non-randomized controlled trials (e.g., before/after and time-series), observational studies, and cross-sectional studies. Publications will be excluded if a single intervention or no intervention was applied, no evaluation was conducted, if they contain no valid measure of frailty or are limited to a study protocol or review of previous studies.

#### Quality appraisal

Studies selected for inclusion will be assessed for risk of bias by two independent reviewers using the *Effective Public Healthcare Practice Project* instrument to assess the quality of quantitative studies [121]. This instrument has been considered suitable for assessment of risk of bias in systematic reviews where randomized and non-randomized study designs were included [122, 123]. Disagreements will be resolved by consensus or adjudication of a third party. Summary scores from the instrument will inform synthesis of information and exploration of heterogeneity in study results [121, 124, 125].

### Data extraction

A data extraction form will be developed and piloted on a sample of included records in Microsoft Office Excel (v. 2016, Microsoft Corporation, Redmond, WA) to ensure adequate capture of characteristics and findings of included studies. One reviewer will extract the data from each primary study independently, then another will verify the accuracy of the extracted data. Disagreements will be settled through discussion with a third author.

The following information will be extracted from each included study:

#### Study characteristics

Author, year, publication type, trial registration number, funding source, setting (country, system of health care, acute/community/primary care), theoretical framing, research question(s), aims of study, design, population, sample, recruitment procedure, outcome measures, test statistics, key findings, limitations as noted by authors and reviewers, conclusions as noted by authors, reviewer notes.

#### Population

Sample size, inclusion/exclusion, number of enrolled and analyzed, description of cases/controls, reasons for withdrawal, missing data, age, proportion female, ethnicity, reason for admission, frailty definition, measure/instrument to identify of frailty, training for frailty instrument application, co-morbidities (dementia, mild-cognitive impairment, known risk for/occurrence of post-treatment delirium, history of falls, occurrence/history of urinary infections/bedsores, other physical health diagnoses, malnutrition), subgroups analyzed in the study.

#### Intervention/strategy

Intervention/strategy name, aim of intervention, intervention description, who delivered the intervention, professionals/others involved, intervention setting, intervention recipient, use of manual/guidelines for intervention, frequency of each intervention component, duration of each intervention component, assessment of fidelity, method of data collection, timing of data collection in relation to intervention, cost of intervention.

### Data analysis

We anticipate a variety of study designs and methods of assessing complex interventions will pose challenges in conducting a meta-analysis. If a meta-analysis is not possible we will pool what data we can, reporting the limitations of our findings. In this case, we will describe findings in a narrative synthesis. To ensure a systematic approach to the narrative synthesis, the guidelines proposed by Cochrane will be adhered to [126]. The narrative will be structured according to study design, type and delivery of interventions, setting, and population. Similarities and differences between findings observed across studies and patterns in data will be outlined. Any data available will be transformed in attempts to find common descriptive and statistical formats for analysis. Within and between study differences will be explored for explanations of direction and size of effects of interventions. Effects of heterogeneity among studies will be discussed, particularly with respect to theoretical framing that may provide explanations for heterogeneity. Finally, the overall strength of the synthesis will be assessed by evaluating the risk of bias results, quality of the evidence, and an overall critical reflection on the synthesis methods [126].

If there are a sufficient number of similar interventions, effects will be pooled using random effects meta-analysis in RevMan (RevMan 5.3 Cochrane). Heterogeneity will be assessed both qualitatively to assess if meta-analysis should be used and measured formally using the *I*-squared statistic if possible with publication bias assessed using funnel plots. If meta-analysis is used, the following subgroup analyses will be considered: age, setting (e.g., community-based care vs. acute care, teaching vs. community hospitals), and acute ward types (e.g., ICU, medicine, surgical, specialist geriatric units, outpatient, emergency department).

## Discussion

Frailty is a relatively new term, and describes both the state of exaggerated vulnerability associated with age-related deficit accumulation, and the associated multidimensional syndrome. To be acceptable to clinicians and the patients they serve, frailty must meaningfully inform decisions about care. As the population ages, health care systems face growing numbers of patients with frailty who may derive less benefit or even more harm from aggressive interventions and invasive procedures while adding cost across the system [127, 128]. In present circumstances, where specialist geriatrician resources are limited, the entire health care workforce needs to be empowered with valid methods of screening for frailty and delivering bespoke models of care [129]. Frailty inclusive care pathways may provide useful guidance in the care of older patients living with frailty in hospital, intermediate and community settings, where clinicians may benefit from presentation of precise considerations in specific patient populations (e.g., primary care, emergency department, surgery) who transition between care settings. Consistent frailty screening and application of frailty-inclusive interventions through existing care pathways and routine care may provide meaningful context to all associated decisions and care provided for this vulnerable population.

### Limitations

Despite our aging population and expected increased incidence of frailty across the care continuum, we expect that there will be limited evidence of evaluation of multi-component interventions aimed to improve care and outcomes for people living with frailty. We expanded the search strategy to maximize findings, although results may demonstrate heterogeneous study designs and frailty assessment methods, limiting our ability to pool data and make inferences for practice. Moreover, we did not include patient or public input in our protocol development and may have focused on a limited perspective of frailty inclusive care. Regardless of the potential limitations we are committed to finding evidence of frailty pathways with rigorous evaluation to inform future practice where possible.

## Conclusions

Frailty-inclusive care interventions that can be incorporated into existing pathways for any disease or clinical setting are an essential part of care continuity for people living with frailty. This review will inform future work to develop and implement pathways aiming to improve the care received by this vulnerable population.

## Supplementary Information

**Additional file 1: Additional Table 1**. PRISMA-P (Preferred Reporting Items for Systematic review and Meta-Analysis Protocols) 2015 checklist

## Data Availability

Not applicable.

## Abbreviations

CDSR: Cochrane Database of Systematic Reviews
CFS: Clinical Frailty Scale
CGA: Comprehensive Geriatric Assessment
CINAHL: Cumulative Index to Nursing and Allied Health Literature
EMBASE: Excerpta Medica Database
ICTRP: International Clinical Trials Registry Platform
ICU: Intensive care unit
MEDLINE: Medical Literature Analysis and Retrieval System Online
MeSH: Medical Subject Headings
PRISMA-P: Preferred Reporting Items for Systematic Reviews and Meta-Analyses Protocols
PROSPERO: International Prospective Register of Systematic Reviews
SCN: Strategic Clinical Network
WHO: World Health Organization
